# Laparoscopic extravesical vesicovaginal fistula repair: our technique and 15-year experience

**DOI:** 10.1007/s00192-014-2458-y

**Published:** 2014-07-16

**Authors:** John R. Miklos, Robert D. Moore

**Affiliations:** 1 International Urogynecology Associates, Atlanta, GA USA; 2 International Urogynecology Associates, Beverly Hills, CA USA; 3 3400 Old Milton Parkway C330, Alpharetta, GA 30005 USA

**Keywords:** Bladder fistula, Laparoscopic vesicovaginal fistula repair, O’Conor, Omental flap, Vesicouterine fistula, Vesicovaginal fistula

## Abstract

**Introduction and hypothesis:**

Two types of laparoscopic vesicovaginal fistula (VVF) repairs, the traditional transvesical (O’Conor) and extravesical techniques, dominate the literature. We present our 15-year experience of primary and recurrent cases of VVF utilizing an extravesical technique, which we first described in 1999.

**Methods:**

An IRB approved retrospective study revealed 44 female patients with either primary or recurrent VVF. Laparoscopic extravesical repair was performed without an omental flap in the majority of cases. A three-layer closure technique was performed utilizing a double-layer bladder closure and a single-layer vaginal closure followed by bladder testing. A suprapubic catheter was utilized for 2–3 weeks postoperatively for bladder decompression.

**Results:**

A review of our experience reveals a 97 % (32 out of 33) cure for primary VVF and 100 % (11 out of 11) rate for recurrent fistulas, with an overall cure rate of 98 % (43 out of 44) at a mean follow-up of 17.3 months (range 3–64). An omental flap was not utilized in 98 % of patients (43 out of 44), with a success rate of 98 % (42 out of 43). The mean estimated blood loss was 39 mL (range 0–450), mean hospital stay was 1.1 days (range 1–3), and none of the patients suffered any major intra- or postoperative complications. None of the patients required a conversion to open laparotomy.

**Conclusions:**

Based upon our experience we believe that performing laparoscopic extravesical VVF repair using a three-layer closure technique without an interposition omentum is a safe, effective, minimally invasive technique with excellent cure rates in an experienced surgeon’s hands.

## Introduction

Surgical repairs of vesicovaginal fistulas (VVF) are most commonly performed: vaginally, abdominally, and laparoscopically. The approach to VVF repair is often dictated by the surgeon’s preference, location or complexity of the VVF. The surgeon’s preference is usually based on his/her training and experience. Our review of laparoscopic/robotic VVF approaches reveals that the most commonly performed approaches are the traditional O’Conor technique and the more recent, less well-known extravesical technique. The O’Conor technique [1] was first described in the 1970s and requires a bladder bivalving technique or cystotomy to identify and repair the VVF. The extravesical technique was first described in the late 1990s [2, 3] and is performed by focusing on a site-specific dissection and repair technique without cystotomy or bivalving of the bladder.

Although there are distinct differences in the two techniques, the literature is quite confusing, often not acknowledging the difference and lumping the two laparoscopic techniques together [4–6], claiming that all laparoscopic techniques are “a variation of the O’Conor technique” [7, 8] or making claims that the laparoscopic extravesical technique is a “novel” technique [9], despite appearing in the literature since the late 1990s [2, 3, 10].

The authors of this paper have described [3, 10–14] and performed the laparoscopic transperitoneal extravesical technique on more than 50 patients with vesicovaginal and vesicouterine fistulas (VUF) over the last 15 years. The goal of this paper is to review our laparoscopic VVF repair experience and to describe and illustrate our laparoscopic extravesical technique.

## Materials and methods

We conducted a retrospective, institutional review board-approved chart review of all patients who underwent a VVF or VUF repair in our practice between January 1998 and January 2014. We identified 48 patients with bladder fistulas, all of whom underwent either a laparoscopic VVF or VUF repair. Forty-four patients had VVF and 4 patients had a VUF repair. All patients with VVF or VUF during this period were repaired laparoscopically and none vaginally or via laparotomy.

Prior to surgical intervention all patients reported their history and underwent a physical examination, a cystourethroscopy, and an intravenous urogram or a computed tomography scan with contrast medium to exclude ureter involvement. All patients’ fistulas were verified at the time of the initial office visit at which office cystoscopy was performed. Patients provided informed consent and specifically were offered continuous drainage via Foley catheter in an attempt at spontaneous closure. We analyzed patients’ charts for age, reason for fistula, previous VVF repair failures, estimated blood loss, hospital stay, and operative complications. Postoperatively, patients were encouraged to come back at either 14 or 21 days and then at 3 months, 6 months, and yearly. They were also encouraged to call if surgical failure was suspected.

**Fig. 1:**
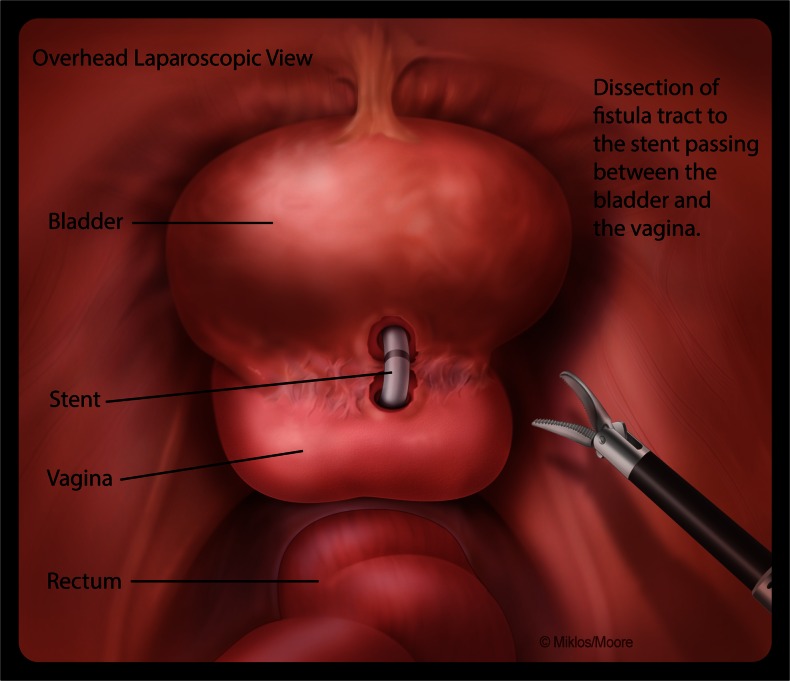
Identification of the ureteral stent traversing the vesicovaginal fistula (VVF) and confirming entry into the fistulous tract

**Fig. 2:**
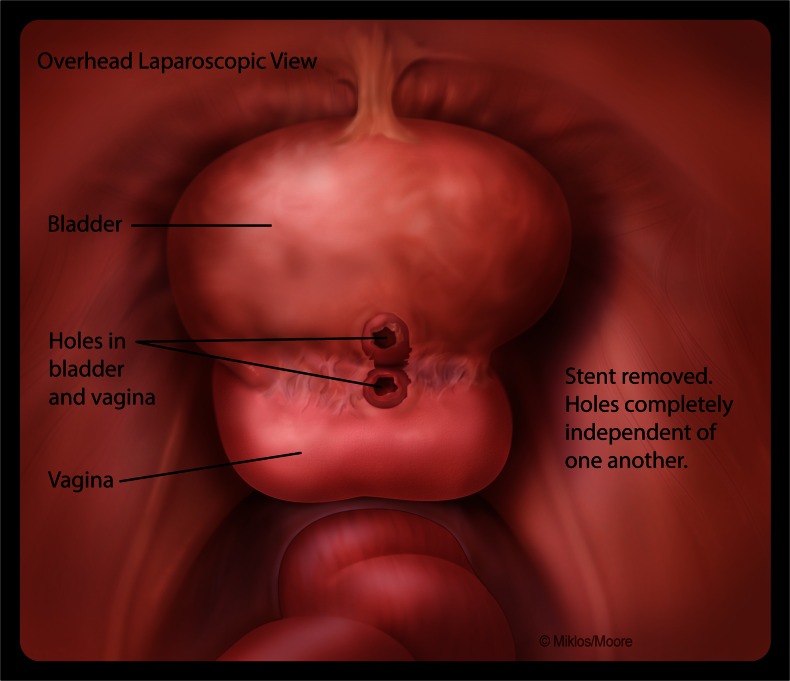
Extravesical VVF dissection with adequate mobilization of the tissue around the tract of both the vagina and bladder

After signing informed consent, patients agreed to a laparoscopic extravesical approach to VVF repair. Cystoscopy was performed and a ureteral stent was placed in the VVF to help identify the fistula at the time of the dissection. Ureteral stents were placed if needed. An open laparoscopy was performed at the inferior edge of the umbilicus where a 10-mm port was placed to accommodate the laparoscope. Three other ports were placed under direct vision. The bladder was retrograde filled with normal saline until the vesicovaginal reflection could be adequately identified. The vesicovaginal space was dissected using endoscopic scissors. The surgeon’s hand was first used in the vagina to help identify the fistula stent and then an end-to-end anastomosis (EEA) sizer was placed to allow a firm backstop during dissection between the bladder and the vagina. Laparoscopic identification of the ureteral stent traversing the VVF confirmed entry in to the fistulous tract (Fig. 1). The stent was then removed and the fistula tract noted in both the bladder and vagina. The tract was excised from both the vagina and bladder and dissection was continued approximately 1–2 cm distal to the site, which allowed for a complete separation of the bladder, the vagina, and the newly excised fistula (Fig. 2).

**Fig. 3:**
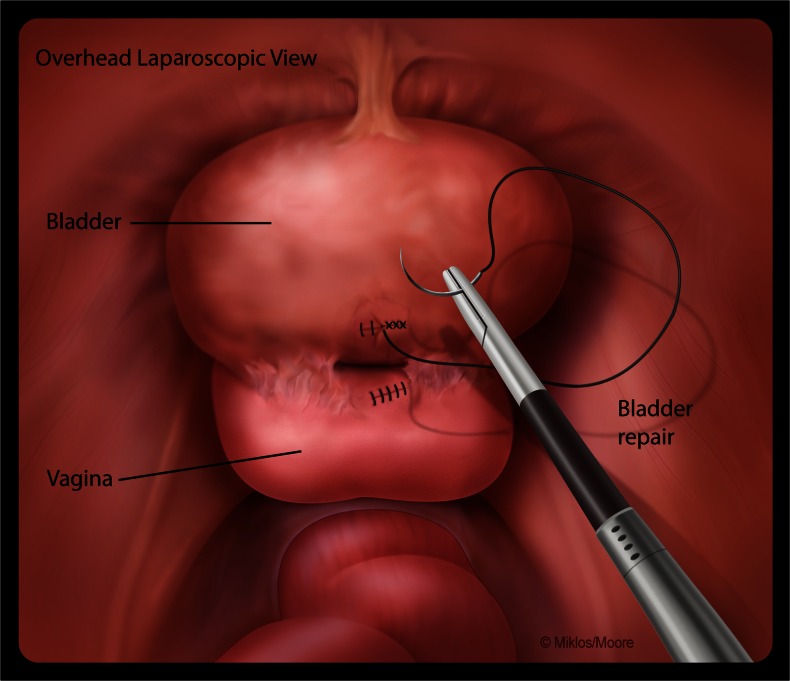
A single-layer closure of the vagina and a double-layer closure of the bladder using a delayed absorbable suture

After adequate dissection and resection of the fistula tract from both the vagina and the bladder, a multi-layered closure was performed. A single layer of 2-0 Vicryl suture was placed in an interrupted figure-of-eight fashion to close the vagina. A double-layer closure using 3-0 Vicryl suture was placed in a figure-of-eight fashion to secure the bladder. After the first layer of closure, the bladder was retrograde filled with 300–400 cc of indigo carmine and sterile water. If a bladder leak was noted the area of weakness was sutured appropriately until no leakage could be verified. After confirming good primary closure of the bladder a second-layer closure was performed using a 3-0 Vicryl suture (Fig. 3). The bladder suture line integrity test was performed again, by filling the bladder with indigo carmine/sterile water solution. All suturing was performed laparoscopically, using extracorporeal knot tying. Cystoscopy was performed after each layer of bladder closure. A suprapubic catheter was placed under laparoscopic and cystoscopic guidance. The laparoscopic ports were removed and all sites were closed. An 18-Fr Foley catheter was placed transurethrally and the patient was sent to recovery with both a suprapubic and transurethral catheter. The transurethral catheter was usually removed within 24–72 h, but only after the hematuria resolved. Patients returned to the office 2–3 weeks postoperatively and an in-office cystoscopy and retrograde bladder fill was performed. If the cystoscopy and the vaginal examination confirmed a successful repair the suprapubic catheter was removed.

## Results

From 1998 until 2014, 48 patients with genitourinary fistula were referred to our center for evaluation and management. Forty-four of these patients were diagnosed and underwent laparoscopic VVF repair. The most common cause of VVF in our case series was hysterectomy (95 %; 41 out of 43) followed by mesh surgery and subsequent erosion in 5 % of the patients (2 out of 43). We were unable to identify the reason for fistula formation in one patient. Cesarean section was the cause of fistula in all VUF patients.

Approximately 25 % (11 out of 43) of the patients in this study had undergone at least one failed previous VVF repair. A total of 17 repairs in 11 patients were recorded: 3 previous repairs (1 patient), 2 previous repairs (4 patients), and a single previous repair (6 patients). Eleven of the surgical failures had occurred after a vaginal attempt at repair, 3 failed previous omentum interposition repairs, 2 of which were via laparotomy and 1 by laparoscopically assisted robotic surgery.

In our study 98 % of the patients (42 out of 43) had a laparoscopic extravesical VVF repair without an interposition graft and 1 patient had the surgery with an interposition graft. The mean age of patients undergoing VVF repair was 46.5 years (range: 31 to 72), estimated blood loss was 51 mL (range: 0 to 450), and mean operative time 144.8 min (range 60–529). The mean time to discharge was 1.2 days (range 0–3 days). There were no serious intraoperative or postoperative complications including: conversion to laparotomy, aborted operative procedure, bowel or ureteral injury, blood transfusion, blood clots, pulmonary embolisms, cardiac events or strokes. Patients were instructed to return to our office 14–21 days after surgery for cystoscopic and vaginal inspection to confirm VVF repair and subsequent suprapubic catheter removal. After a mean of 17.3 months (range: 3–64 months) only 1 patient who underwent a laparoscopic extravesical VVF repair had a recurrence of her fistula, resulting in a 98 % (32 out of 33) cure rate for primary VVFs and 100 % (11 out of 11) for recurrent VVFs. A successful VVF repair was defined as: closed VVF as noted on visual inspection of both the bladder and the vagina, no subjective complaints of vaginal leakage, and no evidence of leakage during Valsalva and cough from the vaginal closure area using a half-speculum for retraction during office cystometry.

## Discussion

The O’Conor transvesical technique was performed via laparotomy for more than 30 years before the first laparoscopic transvesical case was published in 1994 [15]. It was not until 1998 that von Theobold described the first laparoscopic extravesical VVF repair [2]. Von Theobold describes a simple dissection of the bladder away from the vagina and a single-layer bladder closure, as “closure of the vagina was not necessary.” Although a little unorthodox (i.e., a single-layer closure) it was successful in this single case study. An omental J flap was utilized and inserted between the bladder and vagina. A few months later, Miklos et al. [3] described a laparoscopic extravesical technique utilizing a three-layer closure, a double-layer bladder and a single-layer vagina closure, with an intervening omental flap for a patient with recurrent fistula despite two Latzko procedures. Since that time most scientific papers and case studies, focusing on a laparoscopic VVF repairs, have described either a transvesical or extravesical technique.

**Fig. 4:**
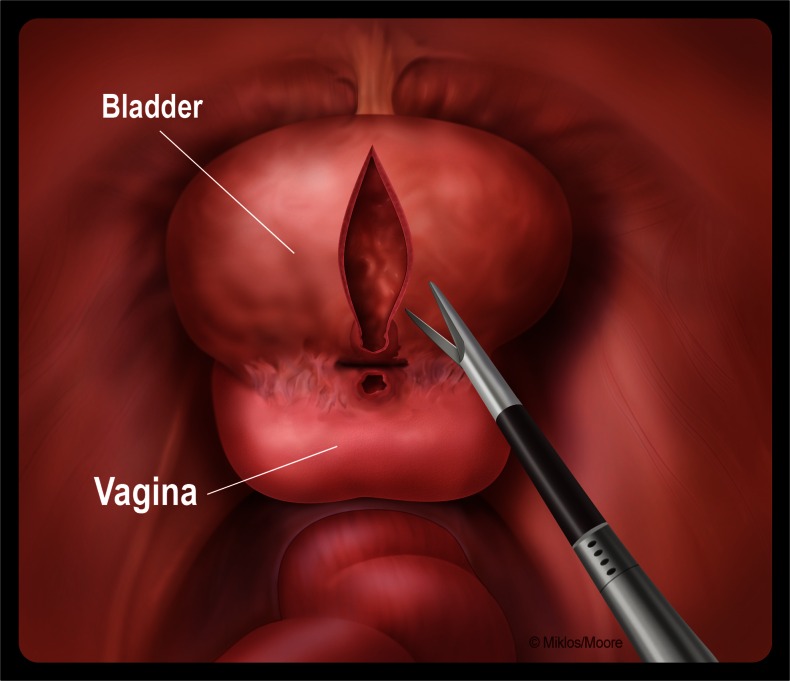
Transvesical (O’Conor) VVF dissection: bivalving with incorporation of the bladder tract and mobilization away from the vaginal tract

Despite the fact that many of these papers describe an extravesical approach, the two procedures are rarely discussed in the same paper, making it difficult to understand the difference. Until recently [16–18], most VVF publications and reviews have neither acknowledged nor distinguished the difference between the transvesical (O’Conor) and extravesical techniques. In fact, some experts have implied that the extravesical technique is a modification of the O’Conor technique [7, 8]. As discussed previously, the traditional O’Conor technique involves a transvesical approach requiring bivalving of the bladder (Fig. 4) [1]. The extravesical approach does not require a cystotomy or a bivalving of the bladder, and therefore is not a modification of the O’Conor technique, but still uses the basic principles of fistula repair, as cited by Couvelaire in the 1950s [19].

The authors believe that the extravesical technique is a less invasive, less traumatic, and possibly a more patient-friendly repair. Using the extravesical VVF site-specific dissection and layered closure technique discussed here, one minimizes the bladder defect by not bivalving the bladder. Bivalving, as in the O’Conor technique, increases the size of the bladder defects and, in theory, increases the chance of failure of the VVF repair. A large incision in the bladder does not increase the success of VVF repair. These two theories can be supported by fistula experts who have stated that there is a greater chance of surgical failure with larger fistulas [20], and attempt to minimize the size of the cystotomy (<2 cm) at the time as an O’Conor technique [21]. Others have reported great success using the nonbivalving extravesical layered-closure technique with and without omental flaps [22, 23].

Although papers written about using interposition grafts in the treatment of VVF are highly suggestive of greater success, definitive proof does not exist. This concept has been debated in the past; most recently, the use of interposition flaps has been questioned in non-irradiated patients [11–14]. In a recent retrospective review of 49 patients without malignancy or a history of radiation therapy the primary surgeon determined that transvaginal repair of benign, recurrent VVFs without tissue interposition can be equally as successful as primary repairs without tissue interposition [24].

An interposition graft for VVFs work on two premises: it functions as a barrier and it introduces vascularity and theoretically lymphatics to improve tissue growth and maturation. It has been the authors’ experience when operating on patients with failed VVFs with omental flaps, upon dissection there was not only a lack of increased vascularity in the area, but there was no evidence whatsoever of an interposition graft. It is our opinion that omental, peritoneal, and sigmoid fat interposition grafts are not as viable as a Martius muscle flap because they lack thickness and vascularity thus minimizing their viability. Omental interposition grafts have never been *proven* to yield a higher cure rate for VVF repairs.

In our series of 43 VVF patients a laparoscopic extravesical repair had a 98 % cure rate without interposition omentum. Our series also includes 11 patients, who had a total of 16 failures, with recurrent VVF, including 3 patients in whom VVF repair failed, despite the use of an omental flap [14]. We also previously reported on a patient in whom 3 previous vaginal surgeries failed who was repaired successfully laparoscopically without an omental flap [13]. All 11 patients with recurrent VVFs were successfully repaired on the first attempt using our described laparoscopic extravesical technique without an omental flap.

The authors attribute their high success rate to meticulous dissection as well as a triple-layer closure, which included a double-layered bladder closure as supported by Sokol et al. [25], as well as aggressive testing of the bladder’s suture line. In a study using 24 mongrel dogs, Sokol et al. suggests that a double-layer closure of cystotomy is superior to a single-layer closure and may prevent fistula. Using a three-layer closure, a double-layer bladder and a single-layer vaginal repair, our study reveals a cure rate of 98 % (43 out of 44).

The authors believe that the only way to determine “good tissue approximation” in VVF repair is to objectively determine a “water tight seal.” Tissue approximation alone without retrograde filling of the bladder and stressing of the suture line is probably not the best measure of suture line integrity. However, the technique to determine a “watertight seal” has never been adequately defined and lacks consistency, as suggested by the literature. The literature suggests that some surgeons use anywhere from 75 cc [21] to 400 cc [14] and others may not perform intraoperative bladder testing at all [16, 17]. Failure to report bladder testing does not necessarily mean it was not done, but based on each published paper we must assume that it was not. The authors of this paper believe that bladder testing is such an important step to VVF repair that it should be recorded and listed as part of each surgeon’s technique. Failure to perform an intraoperative bladder test after a VVF repair is at best careless. It takes little time to perform and if the repair is not watertight it can be reinforced prior to completing the case. Perhaps there is not an absolute volume to instill for a perfect bladder test, but it would certainly make sense to truly test the integrity of the suture line. After all, before attempting a bungee jump you would not test the bungee cord with only a 30-kg sack of sand when some potential jumpers might weigh 150 kg. Why would it be any different when testing a bladder repair? The authors recommend using at least 300–400 cc at the time of bladder fill to test the suture line integrity. This is based upon the normal average bladder capacity of a normally functioning bladder. They also recommend using some type of contrast agent, i.e., povidone or methylene blue, making small leaks easier to see.

Over the last few years we began using another quality assurance measure, which includes placing a white cotton sponge intrabdominally at the suture line and then removing the sponge for closer inspection. If upon removal there is dye on the sponge it encourages us to inspect the suture line more aggressively and repair as needed. It is the surgeon’s responsibility to attempt to minimize failure and these two techniques do not add to morbidity or costs and may just improve the surgical success rate.

Defining the two laparoscopic techniques of laparoscopic VVF repair with and without omental flaps is long overdue as there has been a lack of clarity in the literature. Our technique of laparoscopic extravesical VVF repair is essentially unchanged since we first described the technique in 1999. The only exception is after that case we no longer used omental interposition.

The decision with regard to approach, technique, interposition grafts, and layers of closure remains controversial and remains a personal decision based upon a surgeon’s experience and comfort level. Thus, a surgeon’s decision to approach a VVF vaginally, laparoscopically or via a laparotomy is based primarily on their skill, comfort, and ability. The best technique and surgical approach are those chosen by an experienced surgeon with a specific approach. Vasavada and Raz [26] said it most eloquently: “The best chance for ultimate success of vesicovaginal fistula repair is achieved not only with the first repair, but also the approach most familiar to the surgeon.”

No matter which approach decided upon, the authors believe that the most important aspects of VVF repair remain adequate dissection, a watertight seal, and good postoperative bladder decompression to allow for tissue healing. Our series of laparoscopic VVF repair is currently the largest series in the published indexed literature and suggests that the laparoscopic extravesical technique without an omental flap in non-irradiated tissue is equally as effective in primary or recurrent cases of VVF.
